# Lack of Cathepsin D in the central nervous system results in microglia and astrocyte activation and the accumulation of proteinopathy-related proteins

**DOI:** 10.1038/s41598-022-15805-3

**Published:** 2022-07-08

**Authors:** Chigure Suzuki, Junji Yamaguchi, Takahito Sanada, Juan Alejandro Oliva Trejo, Souichirou Kakuta, Masahiro Shibata, Isei Tanida, Yasuo Uchiyama

**Affiliations:** 1 Department of Cellular and Molecular Neuropathology, Juntendo University Graduate School of Medicine, Bunkyo-ku, Tokyo, 113-8421 Japan; 2 Department of Cellular and Molecular Pharmacology, Juntendo University Graduate School of Medicine, Bunkyo-ku, Tokyo, 113-8421 Japan; 3 Laboratory of Morphology and Image Analysis, Biomedical Research Center, Juntendo University Graduate School of Medicine, Bunkyo-ku, Tokyo, 113-8421 Japan; 4 Division of Morphological Sciences, Kagoshima University Graduate School of Medical and Dental Sciences, Kagoshima-shi, Kagoshima, 890-8544 Japan

**Keywords:** Diseases, Neurology

## Abstract

Neuronal ceroid lipofuscinosis is one of many neurodegenerative storage diseases characterized by excessive accumulation of lipofuscins. *CLN10* disease, an early infantile neuronal ceroid lipofuscinosis, is associated with a gene that encodes cathepsin D (CtsD), one of the major lysosomal proteases. Whole body CtsD-knockout mice show neurodegenerative phenotypes with the accumulation of lipofuscins in the brain and also show defects in other tissues including intestinal necrosis. To clarify the precise role of CtsD in the central nervous system (CNS), we generated a CNS-specific CtsD-knockout mouse (CtsD-CKO). CtsD-CKO mice were born normally but developed seizures and their growth stunted at around postnatal day 23 ± 1. CtsD-CKO did not exhibit apparent intestinal symptoms as those observed in whole body knockout. Histologically, autofluorescent materials were detected in several areas of the CtsD-CKO mouse’s brain, including: thalamus, cerebral cortex, hippocampus, and cerebellum. Expression of ubiquitin and autophagy-associated proteins was also increased, suggesting that the autophagy-lysosome system was impaired. Microglia and astrocytes were activated in the CtsD-CKO thalamus, and inducible nitric oxide synthase (iNOS), an inflammation marker, was increased in the microglia. Interestingly, deposits of proteinopathy-related proteins, phosphorylated α-synuclein, and Tau protein were also increased in the thalamus of CtsD-CKO infant mice. Considering these results, we propose thatt the CtsD*-*CKO mouse is a useful mouse model to investigate the contribution of cathepsin D to the early phases of neurodegenerative diseases in relation to lipofuscins, proteinopathy-related proteins and activation of microglia and astrocytes.

## Introduction

Cathepsin D (CtsD), one of the major lysosomal proteases, is responsible for the degradation of proteins and organelles through the autophagy-lysosomal system^1,2^. The CtsD gene, which encodes cathepsin D, is indispensable for living animals after birth. The gene is identical to the *CLN10* gene, one of the neuronal ceroid-lipofuscinosis (NCL)-causative genes. NCLs are a group of inherited neurogenerative diseases accompanied by lysosomal storage that are classified into at least 14 types^3^. NCLs usually begin in childhood, and are characterized by blindness, myoclonic seizures, progressive motor disturbances, and dementia. The common characteristic in all of these disorders is a striking accumulation of autofluorescent storage materials in all tissues, especially in the central nervous system (CNS).

Whole body CtsD gene knockout (CtsD-KO) mice are born normally but develop a rapidly progressive neurodegenerative disease-like phenotype with seizures, small intestine necrosis, and premature death at approximately postnatal day 26^4,5^. The neuropathologic defects of CtsD-KO mice manifest a phenotype that resembles NCLs^5–7^. In the brain of CtsD-KO mice, the accumulated storage material appears in membrane-delimited organelles with the structure and staining characteristics of ‘residual bodies’, the presumed end result of lysosomal degradation in the cell^8,9^. The storage material contains subunit c of mitochondrial ATP synthase, indicating that the autophagy-lysosome system is impaired^5^.

The autophagy-lysosome system delivers cytoplasmic components into lysosomes via the autophagosome for degradation^10^. Inhibition of the autophagy-lysosome system results in the accumulation of aggregates from autophagy related proteins like ubiquitin, p62, and Nbr1^11,12^. This accumulation of aggregates is a common feature in neurons of different CNS-autophagy deficient mouse models^13–15^.

Microglial activation is considered a key event in neurodegenerative diseases^16–18^. Microglia and astrocyte immunoreactivity are increased in the brain of NCL patients^7^ and conventional CtsD-deficient mice^19,20^. Microglia are normally present as ramified cells that have small cell bodies with numerous branching processes. Once fully activated, ramified microglia have phagocytic activity and are morphologically transformed into cells with large cell bodies with few processes^20^. Inducible nitric oxide synthase (iNOS) and nitric oxide production by microglia is thought to contribute to the neuroinflammatory processes of neurodegenerative diseases^21–23^. In the brain of CtsD-KO mice, microglia express iNOS^20^. In NCLs, upregulation of glial fibrillary acidic protein (GFAP), an astrocyte marker, is also a prominent first pathological sign.

CtsD also contributes to the degradation of α-synuclein and tau^24–26^. CtsD plays major role in cleavage and activation of prosaposin. Recently, prosaposin variants have been linked to Parkinson’s disease^27,28^. An increase in aggregates of pS129-α-synuclein (phosphorylated α-synuclein at Ser129) has also been implicated in the pathogenesis of Parkinson’s disease (PD). In the case of Alzheimer’s disease (AD), an association with the accumulation of hyper phosphorylated tau (phosphatase-sensitive paired-helical-filament-tau, PHF-Tau) has been reported^29,30^. Considering these observations, it seems likely that CtsD-deficiency in the CNS results in the accumulation of pS129-α-synuclein and PHF-tau according to the progression of neurodegeneration.

CtsD is widely distributed in various mammalian tissues^31^. Whole body CtsD-KO mice show NCL-like phenotypes and also exhibit defects not only in the CNS, but also in the small intestine, spleen, and thymus. Therefore, it is likely that a CNS-specific CtsD-deficient mouse model is more suitable to investigate neurodegeneration caused by CtsD-depletion. Our main question in this study is to understand the relationship between cathepin D deficiency and neurodegeneration. To clarify the neuropathological importance of CtsD in the CNS, we generated a CNS-specific CtsD-conditional knockout mice (CtsD-CKO) and found that CtsD in the neurons is indispensable for maintaining viability of neurons in the brain. Additionally, we found significant increases in some proteinopathy-related proteins in the CtsD-deficient brain.

## Results

### CNS-specific CtsD*-*conditional knockout mice developed seizures and exhibited stunted growth

**Figure 1:**
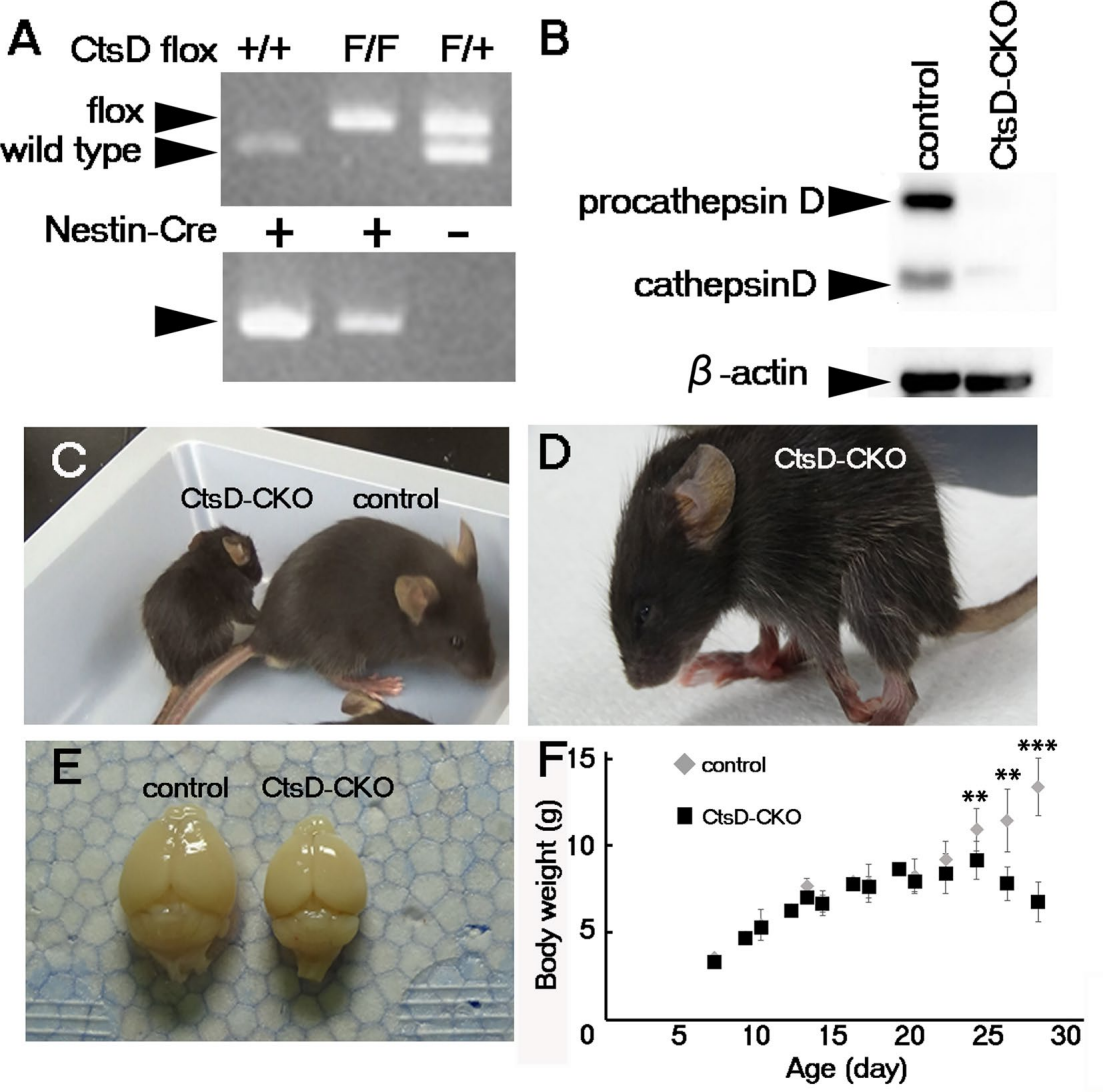
CtsD-Nes mice show stunted growth and neurological dysfunction. (**A**) Genotyping of CtsD-CKO (CtsD^flox/flox^; Nestin-Cre) mice. “F” in the upper panel indicates CtsD-floxed alleles (about 800 bp), and “+” indicates wild type alleles (about 700 bp). In the lower panel, “+” indicates Nestin-Cre transgenes (about 900 bp). (**B**) Representative image of immunoblotting of CtsD in whole brain lysates from the CtsD-CKO and control littermate mice at p14. Molecular masses of pro-cathepsin D (about 46 kDa) and mature cathepsin D (about 28 kDa) are decreased in CtsD-CKO brain lysates. As a loading control in each well, ß-actin is detected using anti- ß-actin antibody. (**C**–**E**) Physiological findings of a 32-day-old CtsD-CKO mouse. The CtsD-CKO mouse is smaller in size than the control littermate mouse (**C**), and shows a kinetic epilepsy-like phenotype (**D**). The size of the brain of CtsD-CKO mouse was smaller than that of the control littermate mouse (**E**). (**F**) Growth defect of CtsD-CKO mice (**p < 0.03, ***p < 0.001, n = three to eleven mice from each genotype mouse.). "Control" indicates CtsD^flox/flox^ mouse, and “CtsD-CKO” indicates CtsD^flox/flox^; Nestin-Cre mouse. Supplementary Fig. 7 shows uncropped PCR banding patterns and blots.

Whole CtsD-KO mice show defects in the small intestine, thymus, and spleen in addition to the CNS tissue^4,5^. To elucidate the role of CtsD in neurons, we generated CNS-specific CtsD-conditional knockout mice by crossing a CtsD^*flox/flox*^ mice with a nestin*-Cre* transgenic mice, which are often used for conditional loss of function studies in the CNS. In the CtsD^*flox/flox*^; Nes-Cre^+^ (*CtsD*-CKO) mouse, the CtsD gene becomes inactivated within the CNS by embryonic day 15.5^32^. Genotyping of CtsD-CKO mouse tails indicated the presence of CtsD^*flox/flox*^ allele and nestin-Cre transgene in the CtsD-CKO mice (Fig. 1A).

To confirm the absence of CtsD in the CtsD-CKO mouse brain, we performed immunoblot analyses using brain lysates prepared from asymptomatic CtsD-CKO mice from postnatal day 14 (p14). In the control mice, CtsD was identified in the brain as pro-CtsD (about 46 kDa) and mature CtsD (about 28 kDa) using anti-CtsD antibody (Fig. 1B). In contrast, pro-CtsD and mature CtsD were hardly detected in the brains of CtsD-CKO mice (Fig. 1B).

Phenotypically, CtsD-CKO mice are born and grow normally until around 3-weeks of age. Around p23, the mice develop seizures and their growth is stunted. At p28, the body weight of the CtsD-CKO mouse is about half of the control mice (Fig. 1C,F). The size of the brain in CtsD-CKO mice is smaller than control mice (Fig. 1E). Finally, CtsD-CKO mice show difficulty to maintain posture (Fig. 1D). Patients with neuronal ceroid lipofuscinosis (NCL) suffer from seizures, physical incapacitation and premature death. This phenotype closely resembles the phenotype of CtsD-CKO mice. These results indicated that lack of CtsD in the CNS causes a severe pathological phenotype that impacts brain size and body weight.

### CtsD-deficiency in the CNS causes the accumulation of autofluorescent lipofuscins, vacuolar autophagosome-like structures, and granular osmiophilic deposits

**Figure 2:**
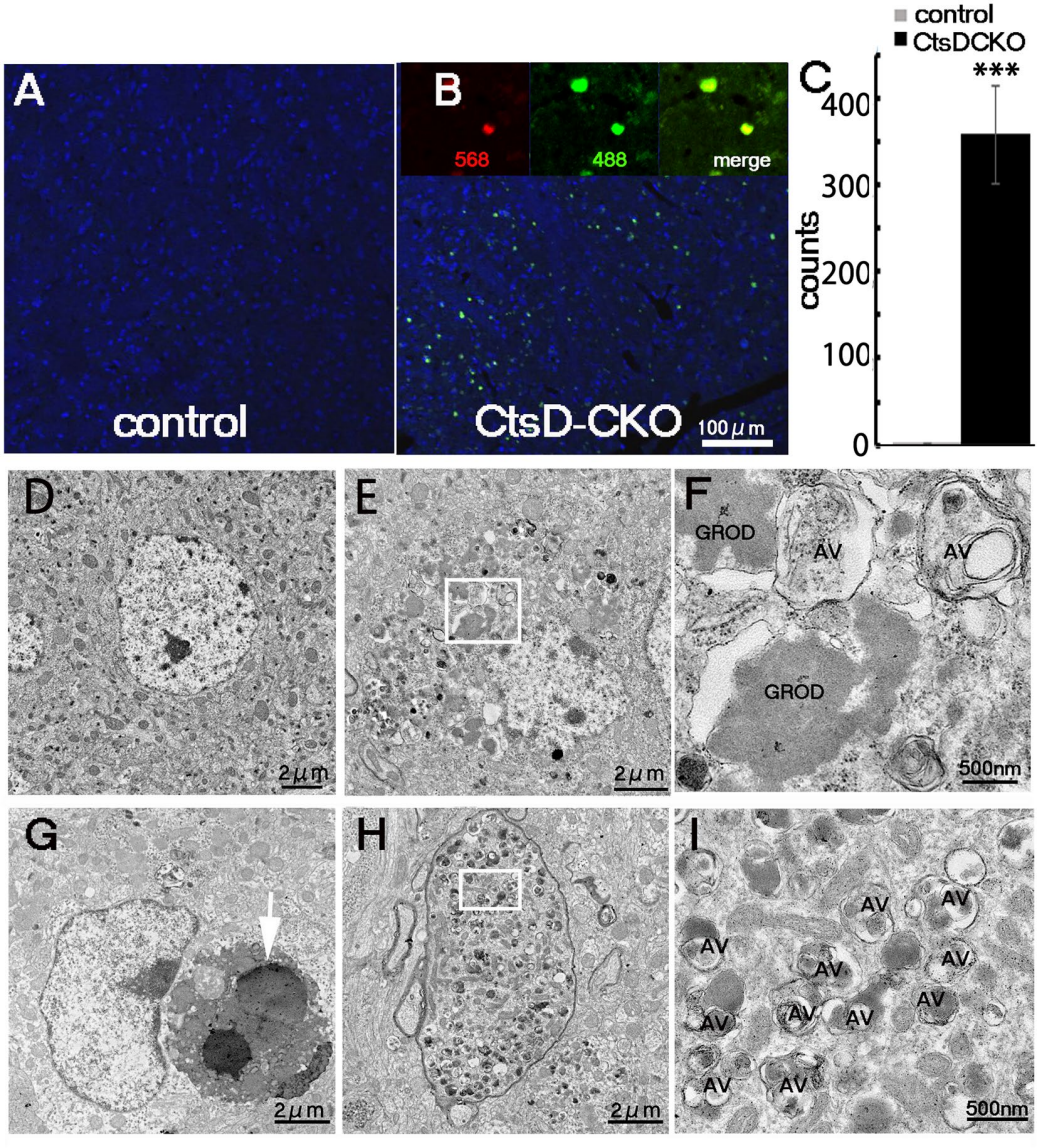
Accumulation of autofluorescent materials in the thalamus of CtsD-CKO mice. Representative images of lipofuscin autofluorescence in the thalamus of CtsD-CKO mice at p23 is shown in (**B**), as compared with that in control mice (**A**). Insets in (**B**) are higher magnified images of autofluorescence by confocal microscopy using light at 568 and 488 nm for excitation. “Merge” is a merged image of the two (568 and 488). Autofluorescent puncta in the thalamus were quantified. (***p < 0.001, from p19 to p25, n = 3 sections form separate mice) (**C**). Representative electron microscopic images of control (**D**) and CtsD-CKO mouse thalami (**E**–**I**) at p25 are shown. (**E**) and (**F**) show representative images of neurons in CtsD-CKO mouse thalamus, which contains numerous dense bodies, resembling granular osmiophilic deposits (GROD), and vacuolar/autophagosome-like structures (AV). A phagocytic cell possesses a degenerated flatten nucleus (white arrow) (**G**). (**H**) and (**I**) show a spheroid-like axon containing numerous AVs and with thinner myelin. (**F**) and (**I**) are higher magnification images of the boxes in respective (**E**) and (**H**).

In the brain of NCL-patients, the accumulation of autofluorescent lipofuscins is observed with a fluorescence microscopy. We first investigated the accumulation of autofluorescent lipofuscins in the brain of CtsD-CKO mice. Autofluorescent signals were clearly observed in the cerebral cortex, hippocampus, and cerebellum of CtsD-CKO mice at p25, while these were hardly recognized in the corresponding regions of the littermate control mice (Fig. 2A,B, Supplementary Fig. 1). Noticeably, autofluorescent signals significantly accumulated in the thalamus of CtsD-CKO mice (Fig. 2C). These results coincided with the previous reports from observation of brains from whole CtsD-KO mice^20^.

As far as we observed, under normal conditions, CtsD-CKO mice showed no histological abnormalities in the small intestine, spleen, or thymus (Supplementary Fig. 2). In contrast, in whole body CtsD-KO mice, the aforementioned pathological findings are observed as part of the phenotype.

We investigated further the morphological changes in the neurons of CtsD-CKO mouse brain by electron microscopy (Fig. 2D–I). Ultrastructural analysis of the brains of NCL-patients have revealed that abnormal structures, so called “GRODs (granular osmiophilic deposits)”, accumulate^33^. We focused on the thalamus of CtsD-CKO mouse brain because the massive accumulation of lipofuscins could be detected in this area (Fig. 2B). Electron microscopic analyses of CtsD-deficient thalamic neurons at p25 revealed that GROD accumulated in the perikaryal region of neurons (Fig. 2E,F; GROD) (Fig. 2D). Brains of NCL10 patients accumulate abnormal autophagosome/autolysosome-like structures, because cathepsin D-deficiency leads to defects in the autophagy-lysosome system. Multi-membranous vacuolar/autophagosome-like structures (AVs) were also observed in the neurons of CtsD-CKO mice (Fig. 2F,I; AV). Phagocytic cells that possessed a degenerated cell with a shrunken nucleus and degenerating cytoplasmic organelles that may be derived from a damaged neuron, were also observed (Fig. 2G). Spheroid-like axons with thinner myelin sheath containing numerous AVs were detected frequently in the thalamus of CtsD-CKO mice (Fig. 2H,I). Accumulation of lipofuscin was detected only in CtsD-CKO mice. These observations show that CNS specific CtsD deficiency can induce the accumulation of abnormal structures including GRODs and AVs similar to the whole body CtsD KO mouse model.

### Ubiquitin- and autophagy-related signals increased in the thalamus of CNS-specific CtsD-conditional knockout mice

**Figure 3:**
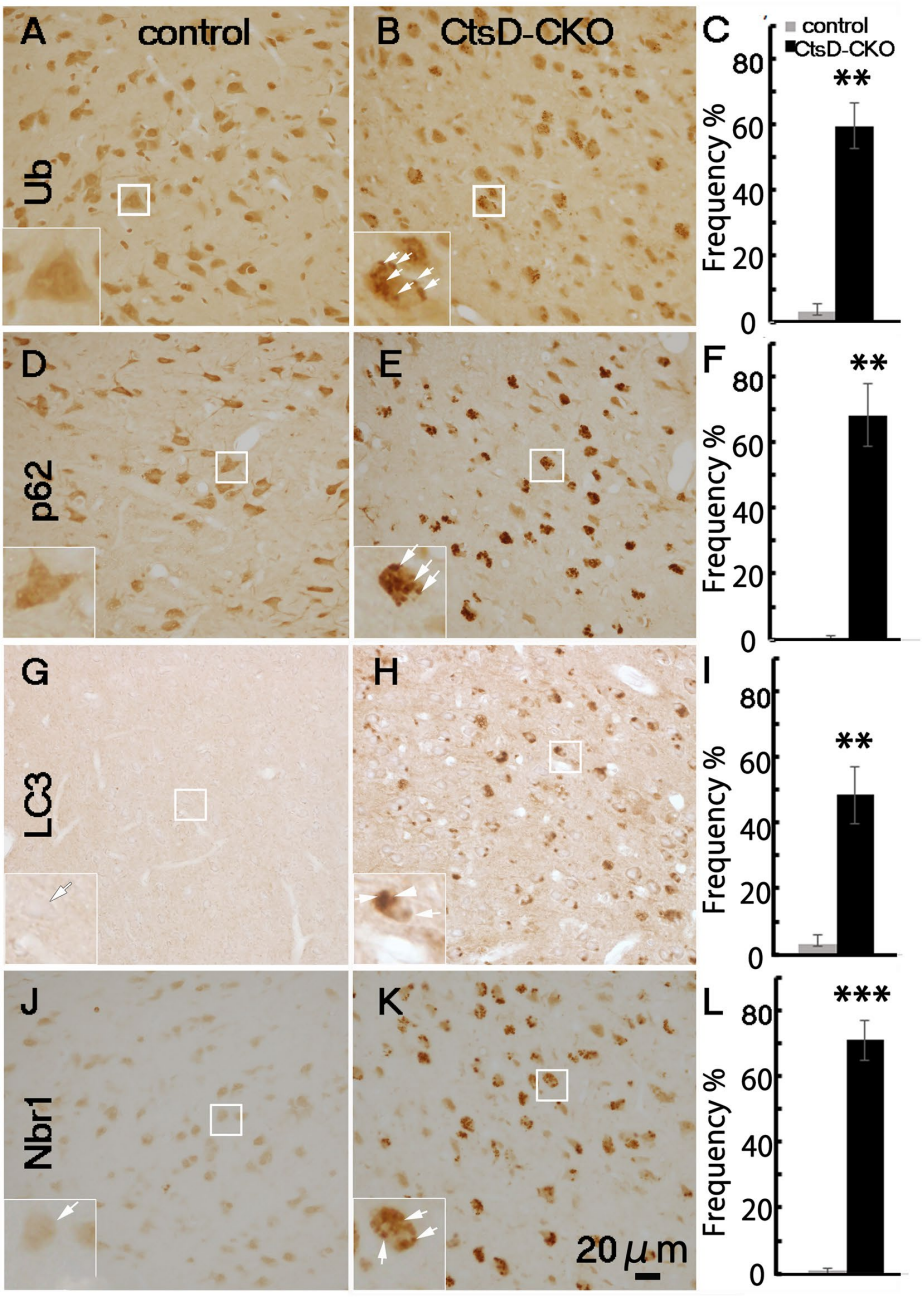
Increase of autophagy-related proteins expression in thalamus of CtsD-CKO mice. Immunohistochemical staining of ubiquitin (Ub) (**A**,**B**), p62 (**D**,**E**), LC3 (**G**,**H**) and Nbr1 (**J**,**K**) in thalami of CtsD-CKO (**B**,**E**,**H**,**K**) and control littermate (**A**,**D**,**G**,**J**) mice at p25. Insets show enlarged boxed areas of neuronal cells in the thalamus of CtsD-CKO that possess immuno-positive granules (arrows). Scale bar; 20 µm. The numbers of cells possessing ubiquitin (**C**), p62 (**F**), LC3 (**I**) and Nbr-1 (**L**) positive granules in the thalamus were quantified. Immunostained brain sections form CtsD-CKO mice were compared with age-matched control littermate mice. (**p < 0.03, ***p < 0.001, from p19 to p25, All data were obtained from three sections form 3 separate mice. Over 72 cells per each section were estimated).

In many cases of patients of neurodegenerative diseases including CLNs, PD, and AD, ubiquitin-positive signals and autophagy-related proteins (p62, Nbr1, LC3) accumulate in the brain. In other mouse models, it has been shown that when the autophagy-lysosome system is impaired in the CNS, positive signals from ubiquitin and p62, a classical receptor of autophagy, are increased^11,12^. Therefore, we performed immunohistochemical analyses of the thalamus of CtsD-CKO mice using anti-ubiquitin and anti-p62 antibodies. As expected, ubiquitin-positive and p62-positive signals increased in CtsD-CKO mice at p25 when compared with those of the littermate control (Fig. 3A–F). Quantification of the neurons containing immuno-positive granules in the thalamus of CtsD-CKO and control mice at p19 to p25 revealed massive accumulation of ubiquitin and p62 (Fig. 3C,F). Signals from Nbr1, a stress inducible protein that cooperates with p62, also increased in *CtsD*-CKO mice (Fig. 3J,K,L).

LC3 is an autophagosomal and autolysosomal marker in mammalian cells^34^. Electron microscopy of neurons in the thalamus of CtsD-CKO mouse revealed an abnormal accumulation of AVs. Therefore, it is possible to think that the LC3-positive signals are increased in the CtsD-CKO mouse neurons according to an increase in AVs. Immunohistochemical analyses with anti-LC3 antibody indicated that LC3-positive signals increased in the thalamus of CtsD-CKO mice, though these were hardly detected in control littermates (Fig. 3G–I). Together, these results suggest that the autophagy-lysosome system was severely impaired in the thalamus of CtsD-CKO mice.

### Microglia and astrocytes are activated in the thalamus of the CNS-specific CtsD-knockout mouse

Activated microglia and reactive astrocytes develop during CNS neuronal damage. These types of glial cells also increase in number in the brains of patients suffering from NCL with the CLN10 gene defect^7^. Considering the neurodegenerative phenotype exhibited by CtsD-CKO mice and the histological abnormality observed in their neurons, it is possible to assume that microglia and astrocytes developed in neurons lacking CtsD. Immunohistochemical analyses of the CtsD-CKO mouse thalamus with anti-CtsD antibody revealed that CtsD-positive cells coincided with activated microglia-like cells, featuring round cell bodies with thick processes (Supplementary Fig. 3. Inset, arrowheads). In general, in flox/flox; nestin-Cre mice, the flox allele was popped out in the neurons and astrocytes, but not in the microglia. Considering the results shown above, it is likely that the microglia are increased in number in CtsD-CKO mice.

**Figure 4:**
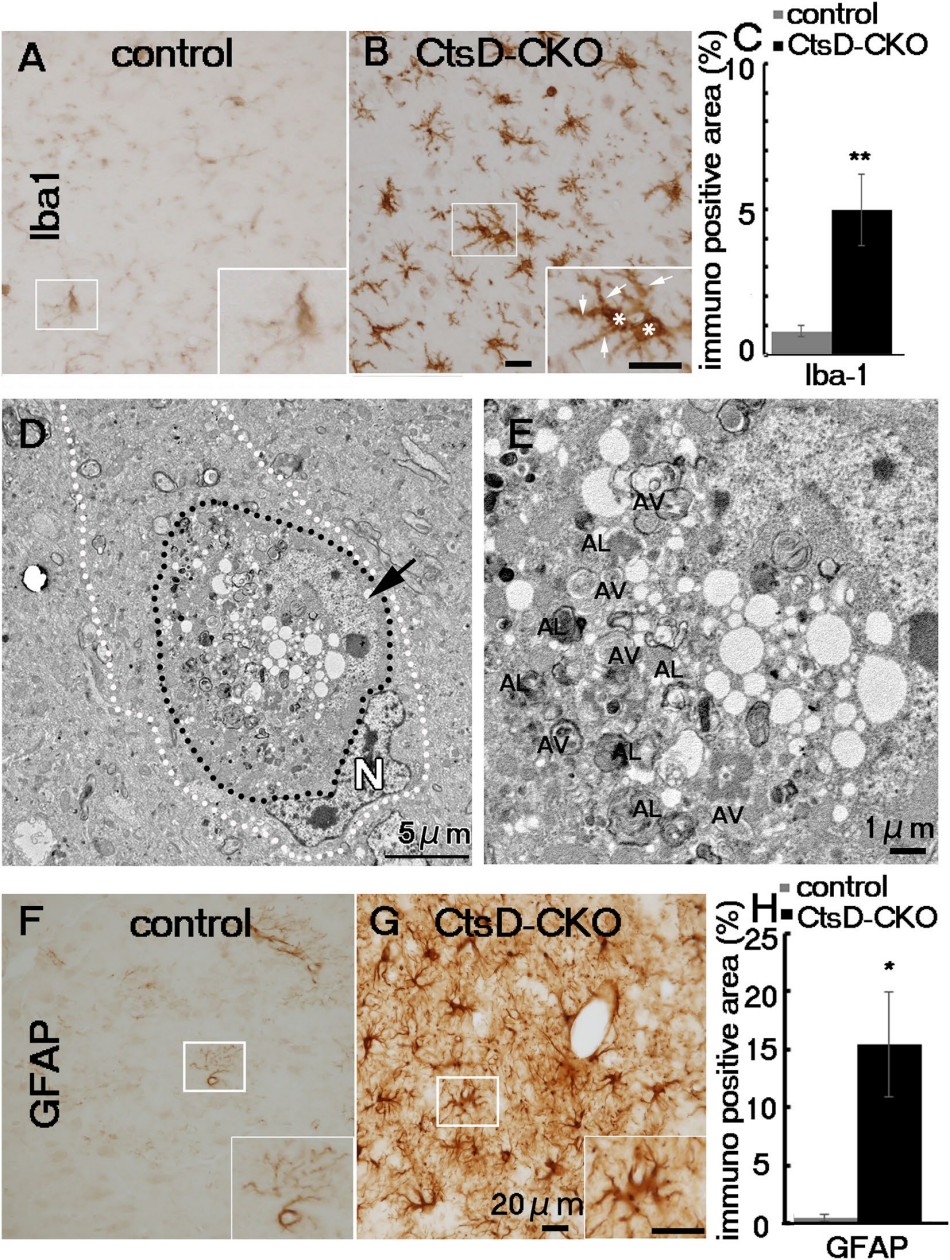
Increase in microglia and astrocyte expression in CtsD-CKO mouse thalamus. Immunohistochemical staining of microglia (Iba1; **A**,**B**) in thalami of CtsD-CKO and control mice at p25. Insets in (**A**) and (**B**) shows a magnified image of the respective boxed area. Arrows indicate thick processes; asterisks indicate expanded round cell bodies. Scale bar: 20 µm. (**C**) Iba1-positive areas in the thalamus of CtsD-CKO mice were compared with age-matched control littermates (**p < 0.03, from p19 to p25 mice, n = three sections form 3 separate mice). (**D**) An electron micrograph of a microglia-like cell (surrounded by white dashed lines) contains a neuron-like cell (surrounded by black dashed lines) with a nucleus (N) and numerous vacuolar structures in the CtsD-CKO thalamus at p25. Arrow, an elongated nucleus of an engulfed neuron. (**E**) A higher magnified image of an engulfed neuron in (**D**). Autophagosome (AV) and autolysosome (AL) like structures were accumulated in the neuron. Immunohistochemical staining of astrocytes (GFAP; **F**,**G**) in thalami of CtsD-CKO and control mice at p25. Insets in (**F**) and (**G**) shows a magnified image of the respective boxed area. Scale bar: 20 µm (**H**) GFAP-positive areas in the thalamus of CtsD-CKO mice were compared with age-matched control littermates (*p < 0.05, from p19 to p25 mice, n = three sections form 3 separate mice.).

To investigate the issue of whether the microglia increase in number in the CtsD-CKO thalamus, we performed immunohistochemical analyses of the thalamic regions with antibody against Iba-1, a marker of microglia in the brain. Iba-1-positive signals were detected in CtsD-CKO mice at p25 when compared with control mice (Fig. 4A,B). Because Iba-1 also expressed in peripheral macrophages, to confirm increased Iba-1 positive cells in the thalamus of CtsD-CKO mice were microglia, we performed double immunostaining of TMEM-119, one of the microglia specific markers, and Iba-1. TMEM-119 signals were increased and colocalized with Iba-1 signals in the thalamus of CtsD-CKO mouse at p25 (Supplementary Fig. 5). Therefore, confirming that these cells were microglia and not macrophages. These results indicated that the microglia increased in number in CtsD-CKO mice. Microglia in the thalamus of CtsD-CKO mice exhibited expanded round cell bodies with thick processes, suggesting microglia activation (Fig. 4B). On the other hand, the microglia in control littermates had small cell bodies and processes attached to the cell bodies (Fig. 4A). Quantification of Iba-1-positive cells indicated a fivefold increase in the number of the microglia in the thalamus of CtsD-CKO mice when compared with control mice (Fig. 4C). Electron microscopic analyses showed microglia engulfing a neuron with heavily accumulated autophagosome/autolysosome-like structures (Fig. 4D,E). In control mice, only few activated microglia cells were detected (Fig. 4A,E, Supplementary Fig. 4).

Expression of inducible nitric oxide synthase (iNOS) has been reported to increase in activated microglia and reactive astrocytes in neurodegenerative diseases^35^. To further confirm whether or not microglia in the CtsD-CKO thalamus were activated, we investigated the expression of iNOS. The iNOS-positive signals in the thalamus of CtsD-CKO mice at p25 were significantly increased, while few signals were observed in the control mice (Supplementary Fig. 4A,B). Iba-1- and iNOS-double positive cells were observed in the thalamus of CtsD-CKO mice. Studies have shown that activated microglia produce tumor necrotic factor alfa, TNF-alfa, which is one of the cytokines that play an important role in iNOS-mediated neurodegeneration^36–38^. Therefore, we performed immunostaining of TNF-α in the thalamus of CtsD-CKO mouse. We added the result in Supplementary Fig. 4C,D.

The results showed that TNF-α expressing microglia are increased in the brain of CtsD-CKO mice. Together these results also show evidence that CtsD-CKO mice exhibit a neuroinflammation phenotype.

Next, we focused on astrocytes in the thalamus because astrocytes have been reported to increase during neuronal damage in the brain. Using immunohistochemistry with an antibody against glial fibrillary acidic protein (GFAP), a marker of astrocytes, we confirmed that GFAP-positive signals were significantly increased in CtsD-CKO mice. Also, GFAP-positive cells appeared thick with heavily branched and thick cellular processes when compared with control mice (Fig. 4F,G). In the thalamus of CtdsD-CKO mice, a 15-fold increase in GFAP positive signals was detected when compared with control mice (Fig. 4H). These results indicated that neuronal damage due to CNS-specific CtsD deficiency induces the activation and increase in microglia and astrocyte number in the thalamus of CtsD-CKO mice.

### Proteinopathy-related proteins, phosphorylated α-synuclein and hyper phosphorylated tau accumulated in the CtsD-CKO mouse thalamus

**Figure 5:**
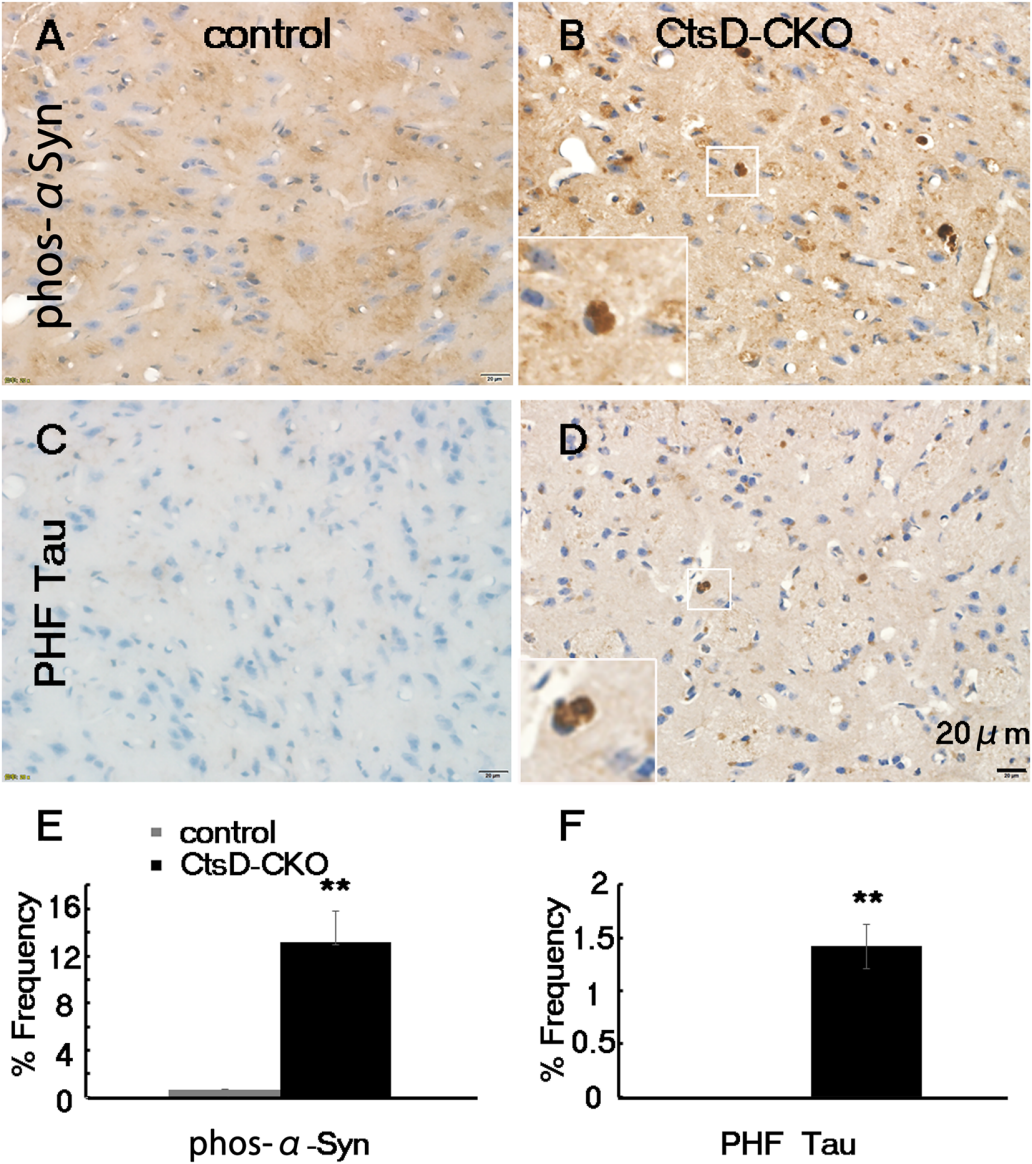
Increase of phosphorylated α-synuclein and hyper phosphorylated tau proteins in the thalamus of CtsD-CKO mice. Immunohistochemical staining of phosphorylated α-synuclein on S129, (phos-α Syn) (**A**,**B**) and phosphatase-sensitive epitope on PHF tau (**C**,**D**) in thalami of CtsD-CKO (**B**,**D**) and control mice (**A**,**C**) at p25. Scale bar: 20 µm. The phosphorylated α-synuclein- and PHF tau-positive cells in the thalamus of CtsD-CKO mice and age-matched control littermates were quantified (**p < 0.03; n = 3 sections form separate mice; Over 120 cells per each section were quantified).

The autophagy-lysosome system contributes to the turnover of intracellular proteins and organelles during aging. Considering that lack of CtsD in the CNS results in the accumulation of lipofuscins, ubiquitins and autophagy-related proteins, it is possible to assume that proteinopathy-related proteins accumulate in the mouse brain of CtsD-CKO mice. To clarify this possibility, we performed immunohistochemistry for pS129-α-synuclein and PHF-tau proteins using specific antibodies in the thalamus of CtsD-CKO mice. pS129-α-synuclein-immunopositive signals were increased in CtsD-CKO mice at p25 when compared with the control mice (Fig. 5A,B). In particular, these immunopositive inclusions for pS129-α-synuclein were observed in the thalamic neurons of CtsD-CKO mice (Fig. 5B). Quantification of the pS129-α-synuclein-positive cells in the thalamus of CtsD-CKO mice revealed a 15-fold increase in pS129-α-synuclein-positive cells when compared with control mice (Fig. 5E).

We also investigated the expression of PHF-tau protein in the thalamus of CtsD-CKO mice by immunohistochemistry. A high number of cells with PHF-tau-positive signals were detected in CtsD-CKO mice, while few PHF-tau-positive cells were detected in control mice (Fig. 5C,D,F). These results indicated that CtsD may play role in preventing proteinopathy-related proteins, pS129-α-synuclein and PHF-tau accumulation. To investigate the neuronal loss and morphological changes in the thalamus of CtsD-CKO mice, we performed Cresyl violet staining (Supplementary Fig. 6). In the thalamus of CtsD-CKO mice, the number of neurons were less than that of control littermates (Supplementary Fig. 6A–C). In CtsD-CKO mice, there were abnormal neuronal cells possessing deeply stained granules, ballooning cytoplasm or ovoid shape cell soma with laterally displaced nuclei (Supplementary Fig. 6E–G), while neuronal cells possessing clear Nissl’s body were observed in control mice (Supplementary Fig. 6D).

## Discussion

In this study, we generated a CNS-specific CtsD-knockout (CtsD-CKO) mouse. In this new mouse model, the neurological symptoms started showing up at around p23. At p25, CtsD-CKO mice exhibited significant accumulation of lipofuscins in the CNS including in the cerebral cortex, hippocampus, cerebellum, and thalamus. Dense bodies resembling granular osmiophilic deposits (GROD), and multi-membranous vacuolar/autophagosome-like structures were observed in the neurons of CtsD-CKO mice. LC3-positive puncta, and cytosolic aggregates containing ubiquitin, p62, and Nbr1 were also increased in the thalamus of CtsD-CKO mice. Together, these results suggest that autophagy-lysosomal pathway is impaired in the neurons. Neurodegeneration is progressive in CtsD-CKO mice, while whole body CtsD-KO mice shows pleiotropic defects in tissues including small intestine, spleen, and thymus in addition to the CNS.

We showed that lack of CtsD in the CNS causes severe defects in brain size and body weight without an apparent defect in the small intestine. CtsD-CKO mice also develop a severe pathological phenotype that manifests as neurological symptoms and progressively exhibit a kinetic epilepsy like phenotype. Their growth is stunted at around p23 and although they do not manifest intestinal symptoms, but at the time of dissection very little amount of food is found in the stomach (data not shown). Therefore, we assume that accumulation of neurological symptoms prevents mice from feeding normally in their critical stage of development, although convulsive attack through neurological disorder cannot be denied.

The expression of CtsD in human brain is high in thalamus during infancy compared to other regions including anterior cingulate cortex, hippocampus, striatum, cerebellar cortex^39^. We think that this could be an indicator that this region has high demand for CtsD. CtsD deficiency results in lysosome degradation dysfunction because CtsD contributes to the activation of other lysosomal hydrolases in addition to proteolysis. Lysosomal dysfunction via CtsD deficiency leads to an excess of autophagosome-formation because, cytosolic contents that include organelles that would be degraded, are accumulated in the cytosol. During autophagy, the autophagosome fuses with the lysosome, and intra-autophagosomal contents are degraded by lysosomal hydrolases. In the thalamus of CtsD-CKO mice, the excess autophagosome will induce excess autophagosome-lysosome fusion. Because of lysosomal dysfunction, the autolysosome (lysosome containing autophagosome) will accumulate in the mice. Finally excess autophagosomes will be unable to fuse with lysosomes, resulting in accumulation of autophagosomes. The accumulation of autophagosomes and autolysosomes in the thalamus of CtsD-CKO mice will be detected as AVs.

It has been reported that AAV vector-mediated CtsD replacement in CtsD-KO mice brain results in an attenuation of neurological symptoms and life extension, while AAV vector-mediated gene transfer of CtsD to the visceral organs of CtsD-KO mice did not lead to any prolongation of their lifespan^40^. These results correspond well to observations in CNS specific CtsD-deficient mice which suffer a premature death. It should be taken into consideration that the difference of AAV gene transfer efficacy is organ-dependent. Considering these results, in the present study we show that CtsD-CKO mice are suitable mouse model for investigating neurodegeneration derived from cathepsin D deficiency and following autophagy-lysosome system impairment.

Similar to suppression of neuron death by re-entry of the CtsD gene to CtsD-KO brains, the prolongation of neuronal survival by inhibition of iNOS production in microglia of whole CtsD-KO brains results in suppression of intestinal necrosis^20^. In our study, CtsD-CKO mice exhibit no obvious abnormality in the small intestine. These results indicate that prolongation of neuronal survival may be important for the suppression of small intestinal necrosis. Moreover, by analyzing the effect of inhibition of iNOS production in CtsD-CKO and whole CtsD-KO mice, it is possible to clarify the issue of whether visceral disorder depends on iNOS production in microglia in CtsD-KO brains or not.

Microglia in the thalamus of whole CtsD-knockout mouse phagocytoses neurons laden with autophagosome–autolysosome-like bodies, and iNOS expression in the microglia was increased^20^. Nitric oxide production via iNOS in microglia and peripheral macrophages contribute to secondary tissue damage^20^. Microglia in the whole CtsD-knockout mice lacks cathepsin D, while microglia in this CtsD-CKO mice has cathepsin D. Considering that iNOS and TNFα were increased even in the CtsD-CKO mouse, it is possible that CtsD deficiency directly causes neuronal damage independent of microglial activation.

Microglia were significantly activated and increased in number in the thalamus of the CtsD-CKO mice. Autophagy controls the inflammatory response in microglia^41,42^. Under physiological conditions, microglia play an important role in neuronal survival by producing neurotrophic factors as well as by phagocytosing dead cells, protein aggregates, cellular debris, and invading pathogens^43^. In some pathological situations, microglia play critical roles in regulating neuronal activity and function^44^. In CtsD-CKO mice, Cre recombinase was expressed in neurons and astrocytes, but not in microglia, in the brain^45^. CtsD was expressed well in the microglia of CtsD-CKO mice (Supplementary Fig. 2). It is likely that the increase in number and size of activated microglia is a consequence of the progressing neurodegeneration in the mouse due to lack of CtsD in the neurons.

Astrocytes are responsible for the maintenance of brain homeostasis, including the regulation of the blood–brain barrier, synaptic function, and glutamate uptake^46,47^. On the other hand, they also play crucial roles in pathological processes, including neurodegenerative diseases, such as AD, PD, stroke and CNS injuries^48,49^. In Batten Disease, known also as infantile neuronal ceroid lipofuscinosis^50^, GFAP upregulation is a highly prominent first pathological sign^51,52^. Similarly, GFAP positive cells were increased in number and cell size in the brain of CtsD-CKO mice.

The expression of inducible nitric oxide synthase (iNOS) and TNFα in microglia and the production of large amounts of nitric oxide are thought to contribute to the neuroinflammatory processes of neurodegenerative diseases such as AD, PD, multiple sclerosis, amyotrophic lateral sclerosis^21–23^. iNOS and TNFα was expressed in Iba1-positive microglia in the brain of CtsD-CKO mice (Supplementary Fig. 3) consistent with the previous study of whole CtsD-knockout mice^20^.

Does glial activation occur before neurodegeneration in the CtsD-deficient brain? Or does neurodegeneration precede glial activation? In whole CtsD-knockout mice, GFAP-positive signals are detected as spots in the thalamus at p16 and further spread throughout the thalamus by p24^53^. In addition, few F4/80 (a marker of macrophage and microglia)-signals are observed in the thalamus of whole CtsD-knockout mice at p16 however, they become evident at p20^53^. The number of neurons in CtsD-KO thalamus was significantly reduced at p20 and p24 but not at p16^53^. Therefore, in whole CtsD-knockout mice, it is possible that activation of astrocytes precedes that of microglia, and glial activation precedes neuronal loss.

Different from the case of whole CtsD-knockout mice, microglia in the CtsD-CKO mice possess cathepsin D. Significant activation of microglia and astrocytes and a decrease in the number of neuronal cells were also observed in the thalamus of the CtsD-CKO mice. It is probable that neuronal damage preceded microglial activation in CtsD-CKO mice brain. However, it also possible that the triggers of microglia activation may be different from those of CtsD deficient microglia in CtsD-KO mice. At present, it is difficult to answer the issue of why microglia in CtsD-CKO mice cannot remove damaged cells or materials without adversely affecting brain environment, and the issue of what triggered microglia enhancing cytotoxicity. Further studies including age-dependent analyses will be required.

Recently, the relation of cathepsin D with neurodegenerative diseases is getting great attention^27,28^. It has been reported that α-synuclein and tau are degraded by cathepsin D^25^. Other study showed that cathepsin D is up-regulated in the brain of AD and PD patients^54^. Also, it was reported that AD and PD patients have nerve cell damage in their thalamus^55^. In CtsD-CKO mice, we also showed evidence that neurons in the thalamus exhibit signs of cell damage. In addition, phosphorylated-α-synuclein and/or PHF-tau-positive deposits accumulated in the thalamus of CtsD-CKO mice (Fig. 5). These results showed that CtsD is a key enzyme that contributes to the degradation of phosphorylated-α-synuclein and PHF-tau among a diverse group of lysosomal enzymes.

Many transgenic mouse models of PD and AD have been reported^56,57^. However, it takes about 8–9 months for these mice to begin to develop pathological abnormalities in the brain. These mice exhibit very few symptoms that are difficult to detect even by behavioral tests. In contrast, the onset of pathological abnormality and neurological symptoms of CtsD-CKO mice were observed at the very early stages of life (around p20). These findings suggest that the CtsD-CKO mouse is potentially a useful mouse model to elucidate the mechanism of accumulation and/or degradation of proteinopathy-related proteins for neurodegenerative diseases such as PD and AD in addition to ceroid-lipofuscinosis.

## Materials and methods

### Animal model

All animal experiments were performed in accordance with the Laboratory Animal Experimentation guidelines of Juntendo University (project license no.290197), carried out in compliance with the ARRIVE guidelines (http://www.nc3rs.org.uk/page.asp?id=1357) and approved by the Institutional Animal Care and Use Committee of Juntendo University. For tissue collection of mice, all mice were anesthetized by an intra-peritoneal injection of a lethal dose of pentbarbital (> 120 mg/kg) followed by cardiac puncture.

The CNS-specific CtsD-knockout mouse was established by breeding CtsD^flox/flox^ mice with Nestin-Cre^+^ transgenic mice (Jackson Laboratories). Generation of CtsD^flox/flox^ mouse was described previously, where the targeted allele of the CTSD gene, exon2, was sandwiched by a pair of loxP^19^. Nestin-Cre^+^ mouse is sufficient for recombination in neural stem cells and intermediate neural progenitor cells^58,59^. Briefly, CtsD^flox/flox^ mice were interbred with Nestin-Cre^+^ mice to obtain CtsD^flox/+^; Nestin-Cre^+^ mice. CtsD^flox/+^; Nestin-Cre^+^ mice were crossed with CtsD^flox/flox^ mice to get CtsD^flox/flox^; Nestin-Cre^+^ and CtsD^flox/flox^ littermates that served as control. Nestin-Cre^+^ carries a promoter segment of Nestin and cDNA encoding a Cre recombinase. Selection of CtsD^flox/flox^; Nestin-Cre^+^ was performed by genomic polymerase chain reaction (PCR). Briefly, template genomic DNA was isolated from tail biopsies and examined by PCR. For the identification of the CtsD-flox allele the following primers were used: CtsDF/F forward: TGGCGTCCCCTATTTAGTTGTCTGCATCAG and CtsDF/F reverse: AAATGGCCACAACATGTCACAAACTCCTGC. For the identification of the Nestin-Cre transgene the following primers were used: Nes forward: TTTGCCTGCATTACCGGTCGATGCAAC and Nes reverse: TGCCCCTGTTTCACTATCCAGGTTACGGA. DNA amplification was performed by C1000 thermal Cycler (Bio Rad.CA). Either sex of mice was used in all experiments.

### Antibodies

Rabbit polyclonal anti-CtsD antibody was produced and purified by affinity chromatography, as reported previously^60–62^. Rabbit anti-LC3 (#12741) and mouse anti-ubiquitin (#3936) antibodies were purchased from Cell Signaling Technology (Danvers, MA). Guinea pig anti-p62 antibody was purchased from PROGEN Biotech GmbH (Heidelberg, Germany, #GP62-C). Rabbit anti-Iba1 (#019-19741) and mouse anti-phosphorylated-α-synuclein (#014-20281) were purchased from FUJIFILM Wako Pure Chemical Corp (Tokyo, Japan). Mouse anti-phosphorylated tau antibody was purchased from Thermo Fisher Scientific (Waltham, MA, #MN1020). Mouse anti-Nbr1 (#ab55474) and rabbit anti-TMEM-119 (ab209064) were purchased from Abcam (Cambridge, MA, #ab55474). Mouse anti-GFAP (#G3893) and mouse anti-β-actin (#A5441) antibodies were purchased from Sigma-Aldrich (St. Louis, MO). Mouse anti-iNOS (# 610329) was purchased from BD Transduction Laboratories. Mouse anti-TNFα (sc-52746) was purchased from Santa Cruz Biotechnology (Santa Cruz, CA). 4′,6-Diamidino-2-Phenylindole (DAPI) was purchased from Thermo Fisher Scientific (#D1306).

### Sample-fixation and Epon-embedding of samples for electron microscopy

For electron microscopy, brains were fixed by intracardiac perfusion of 2% paraformaldehyde and 2% glutaraldehyde buffered with 0.1 M phosphate buffer, pH 7.2^5,63^. Fixed brains were cut into small pieces, dehydrated with a graded series of ethanol, and embedded in epoxy resin (TAAB Epon 812, EM Japan, Tokyo, Japan). For light microscopic observations, semi-thin sections were cut at 500 nm with an UC6 or UC7 ultramicrotome (Ultracut CUT N, Leica, Nussloch, Germany) and stained with toluidine blue. For electron microscopy, thin sections were cut at 70–80 nm thicknesses with the ultramicrotome, mounted on grids, stained with uranyl acetate and lead citrate, and observed with an electron microscope (Hitachi HT7700, Tokyo, Japan).

### Immunohistochemistry

CtsD-CKO mice were perfused through heart with 4% paraformaldehyde, and brains were excised from the mice. The tissues were further fixed in the same fixative and frozen in OCT compound (Tissue-Tek, Torrence, CA) to prepare 8 μm-thick sections. Frozen sections were then washed with a phosphate-based saline, pH 7.2, and quenched with 0.025% hydrogen peroxide in methanol for 30 min followed by immunostaining with antibodies. Sections were further incubated with biotinylated secondary antibodies and reacted with peroxidase-conjugated streptavidin (Vectastain ABC kit, Vecor Laboratories, Burlingame, CA).

For quantification of immunostained samples, 352 × 264 µm^2^ images of mouse brain tissue sections were obtained using an Olympus BX63 microscope. Microscopic fields with over 70 cells per field were used to count the number of cells with immuno-positive signals (n = over 3). For quantification of microglia and astrocytes, respective Iba1 and GFAP positive areas in the same size field were estimated using Image J software (https://imagej.nih.gov/ij/index.html).

### Cresyl violet staining

Cresyl violet acetate (WALDECK; 1A-400) was dissolved in distilled water at 0.1% (w/v) and glacial acid was added to this solution. The sections were stained and dehydrated by a series of different grades of ethanol. For quantification, 352 × 264 µm^2^ images of mouse brain thalamus were taken using an Olympus BX63 microscope. Cell counts were obtained as total cell number from 10 images in each group. Nineteen to thirty-two days old six separate CtsD-CKO brain sections were compared with same age littermate control. The result was expressed as ratio to control.

### Immunoblot analysis

Total lysates were prepared from mice brains using a Polytron homogenizer in a lysis buffer consisting of 0.05 M Tris–HCl, pH 7.5, 0.15 M NaCl, and 1% Triton X-100 with protease inhibitor cocktail (100×) (Nacalai Tesque, Kyoto, Japan, cat# 25955-11). After centrifugation at 20,000*g* for 15 min at 4 °C, the supernatants were collected. The concentration of total proteins in the supernatants was measured using a BCA protein assay kit (Thermo Fisher Scientific). Total proteins were separated by SDS-PAGE using Extra PAGE One precast gels (7.5–15%, Nacalai Tesque cat# 13065-54). Total proteins on the gels were transferred to polyvinylidene difluoride membranes. The membranes were incubated with the primary antibodies at 4 °C overnight. After washing the membranes with TBS-T buffer to remove excess antibody, they were incubated with 0.1% horseradish peroxidase-conjugated secondary antibodies (Thermo Fisher Scientific). Subsequently, HRP-labeled antibodies on the membranes were detected by the enhanced chemiluminescence detection system (ECL Plus Substrate; Thermo Fisher Scientific, cat# 34080) according to the manufacturer’s protocol.

### Statistical analysis

The data obtained from each experiment were expressed as the mean ± SEM. Statistical analyses were performed using Student’s t-test, and statistical significance was set at p < 0.05.

## Supplementary Information

Supplementary Information.
