# Supplementary material for: Lack of Cathepsin D in the central nervous system results in microglia and astrocyte activation and the accumulation of proteinopathy-related proteins
Source: Sci Rep. 2022 Jul 8;12:11662. doi: 10.1038/s41598-022-15805-3 (PMC9270453; doi:10.1038/s41598-022-15805-3)
Supplement: Supplementary file 1 — Supplementary Information. [file 41598_2022_15805_MOESM1_ESM.pdf]

Lack of Cathepsin D in the central nervous system results in microglia and astrocyte activation and the accumulation of proteinopathy-related proteins.

Abbreviated Title: CNS-specific *CtsD*-knockout mice

Chigure Suzuki[1, 2#], Junji Yamaguchi[1, 3#], Takahito Sanada[1], Juan Alejandro Oliva Trejo[1], Souichirou Kakuta[1, 3], Masahiro Shibata[4,] Isei Tanida[1*], Yasuo Uchiyama[1*].

Supplementary information includes:

Supplementary Figs. 1-7

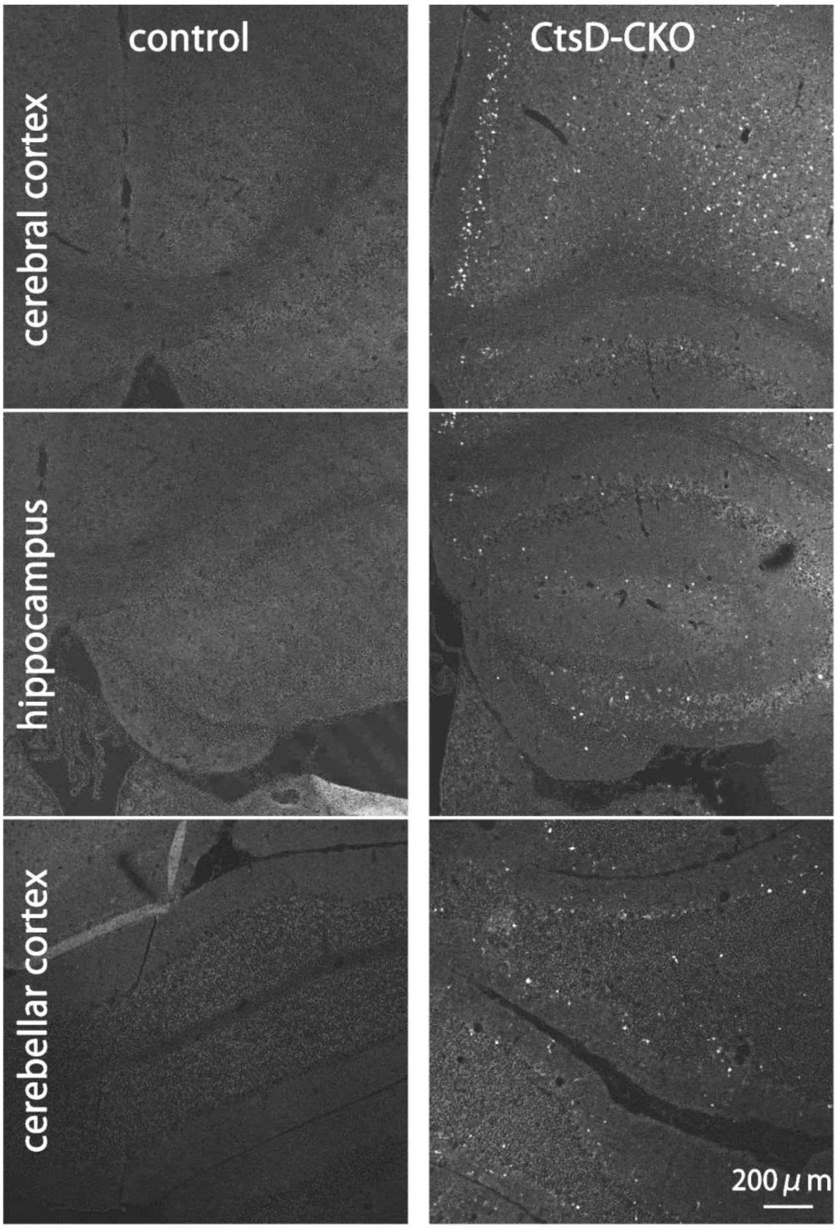

Supplementary Fig. 1. Accumulation of autofluorescent lipofuscins in the brain of CtsD-CKO mouse. Representative images of autofluorescent signals (white particles) in the cerebral cortex, hippocampus, and cerebellum of CtsD-CKO and littermate control mouse brains at p25. Scale bar: 200 µm.

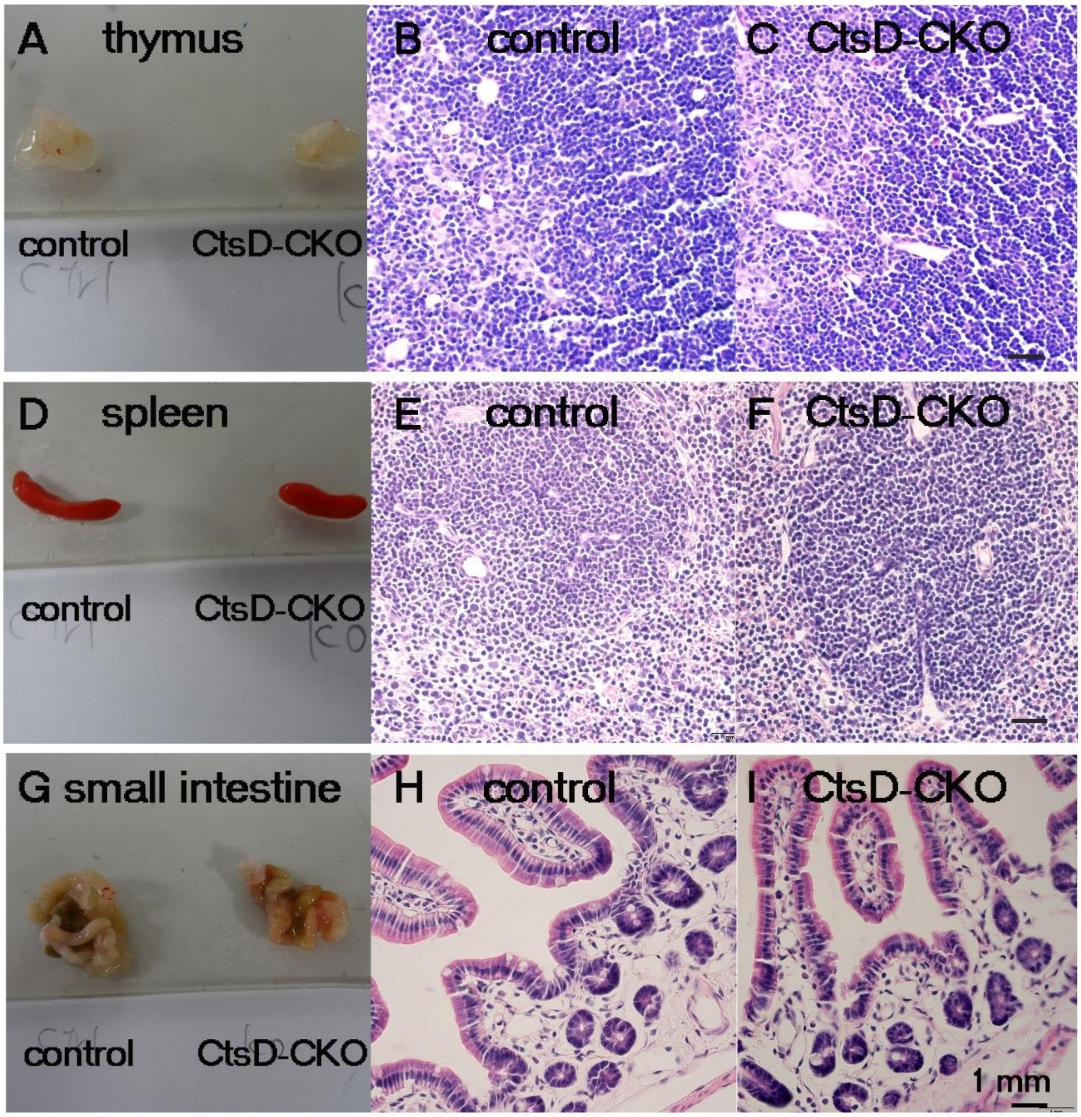

Supplementary Fig. 2. CtsD-CKO mice showed no necrotic injury in their thymus, spleen and small intestine at p25. The size of thymus (A), spleen (D) and small intestine (G) of CtsD-CKO were smaller than those of control littermate at p25. H&E staining of thymus (B: control, C: CtsD-CKO), spleen (E: control, F: CtsD-CKO) and small intestine (H: control, I: CtsD-CKO) tissues showed no apparent histological abnormalities. Scale bar: 1 mm

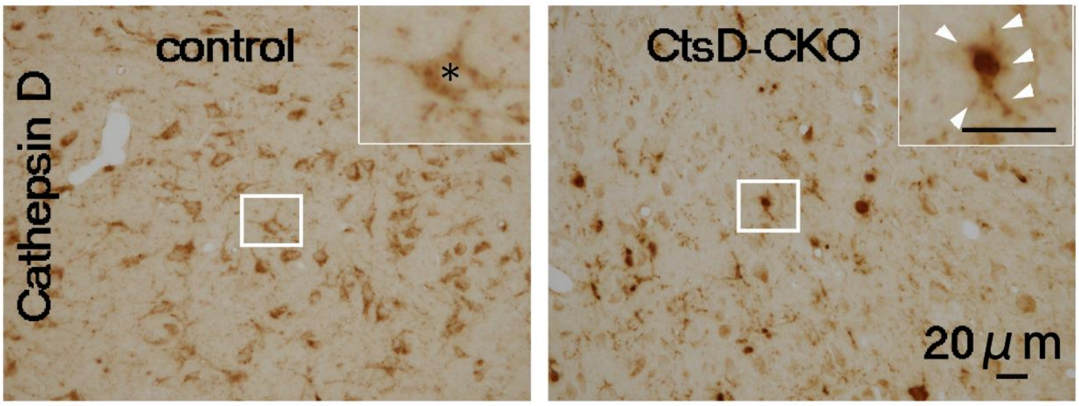

Supplementary Fig. 3. CtsD expression in the thalamus of CtsD-CKO mice and control littermates at p25. Immunohistochemical staining for CtsD was performed in the thalami of CtsD-CKO and control mice at p25. Insets show enlarged images of the boxed areas in the thalami of control and CtsD-CKO mice. Positive staining for CtsD is mainly detected in neurons in the brain of control mice, while the nerve cell body shown in an inset contains a large nucleus (asterisk) and the perikaryal region where densely stained areas could be detected. On the other hand, CtsD-positive staining is densely detected in smaller cells with processes that seem to be microglia in the thalamus of CtsD-CKO mice. Inset shows a microglia positively stained for CtsD exhibiting branching processes (arrowheads). Scale bar: 20 μm.

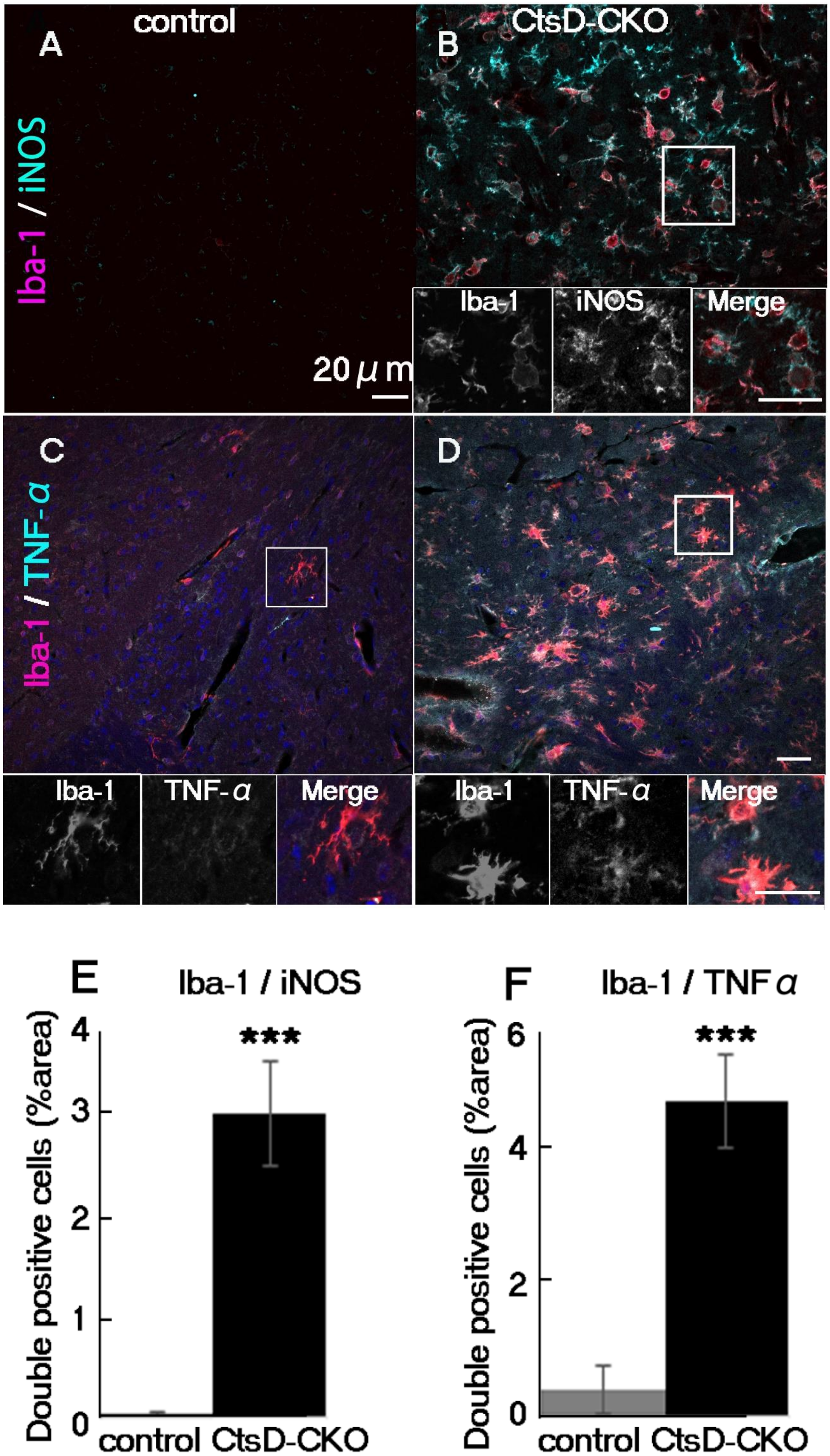

control
CtsD-CKO
A
B
Iba-1 / iNOS
20 μm
Iba-1
iNOS
Merge
C
D
Iba-1 / TNF-α
Iba-1
TNF-α
Merge
Iba-1
TNF-α
Merge
E
Iba-1 / iNOS
***
Double positive cells (%area)
4
3
2
1
0
control
CtsD-CKO
F
Iba-1 / TNFα
***
Double positive cells (%area)
6
4
2
0
control
CtsD-CKO

Supplementary Fig. 4. iNOS- and Iba-1-double positive microglia are abundant in the thalamus of CtsD-CKO at p25. Imunoreactivity for iNOS (Cyan) is hardly recognized in the control thalamus, although weak staining for Iba-1 (Red) is detected dispersedly in this area (A). In the thalamus of CtsD-CKO mice, Iba-1-positive microglia are abundantly detected and also stain positive for iNOS (inset) (B). TNFα (Cyan) and Iba-1 (Red)-double positive microglia are abundant in thalamus of CtsD-CKO (D), but not control littermate mice at p25 (C). TNFα is hardly recognized in the control thalamus (inset in C). In the thalamus of CtsD-CKO mice, Iba-1-positive microglia are strongly overlapping with TNFα (inset) (D). Insets in C and D (Iba-1, TNF, and Merge) show enlarged respective boxed areas. Scale bar: 20 µm. (E) Iba-1- and iNOS- double positive cells per area in A and B was quantified (mean ± SEM, n = 3, ***$p<0.01$). (F) Iba-1- and TNF-α-double positive cells per area in C and D was quantified (mean ± SEM, n = 3, ***$p<0.01$).

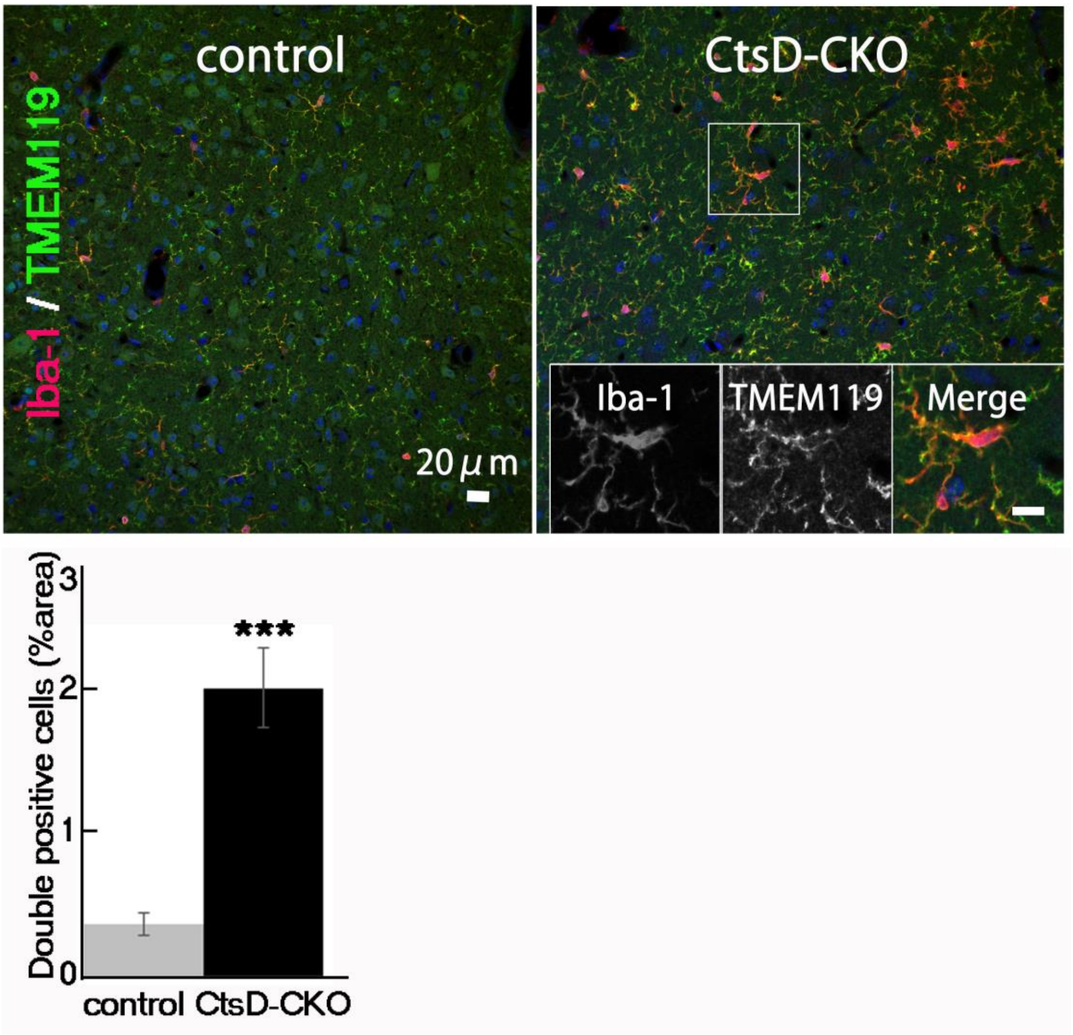

Supplementary Fig. 5. Iba-1 (Red) and TMEM-119 (Green)-double positive microglia are abundant in thalamus of CtsD-CKO at p25. Iba-1-positive microglia (Red) are strongly overlapping with TMEM-119 (Green) in the CtsD-CKO but not in control mice. Inset shows enlarged boxed areas. Scale bar: 20 μm. Graph shows quantification of Iba-1 and TMEM-119-double positive cells per area in **control** and **CtsD-CKO** (mean ± SEM, n = 3, ***p<0.01).

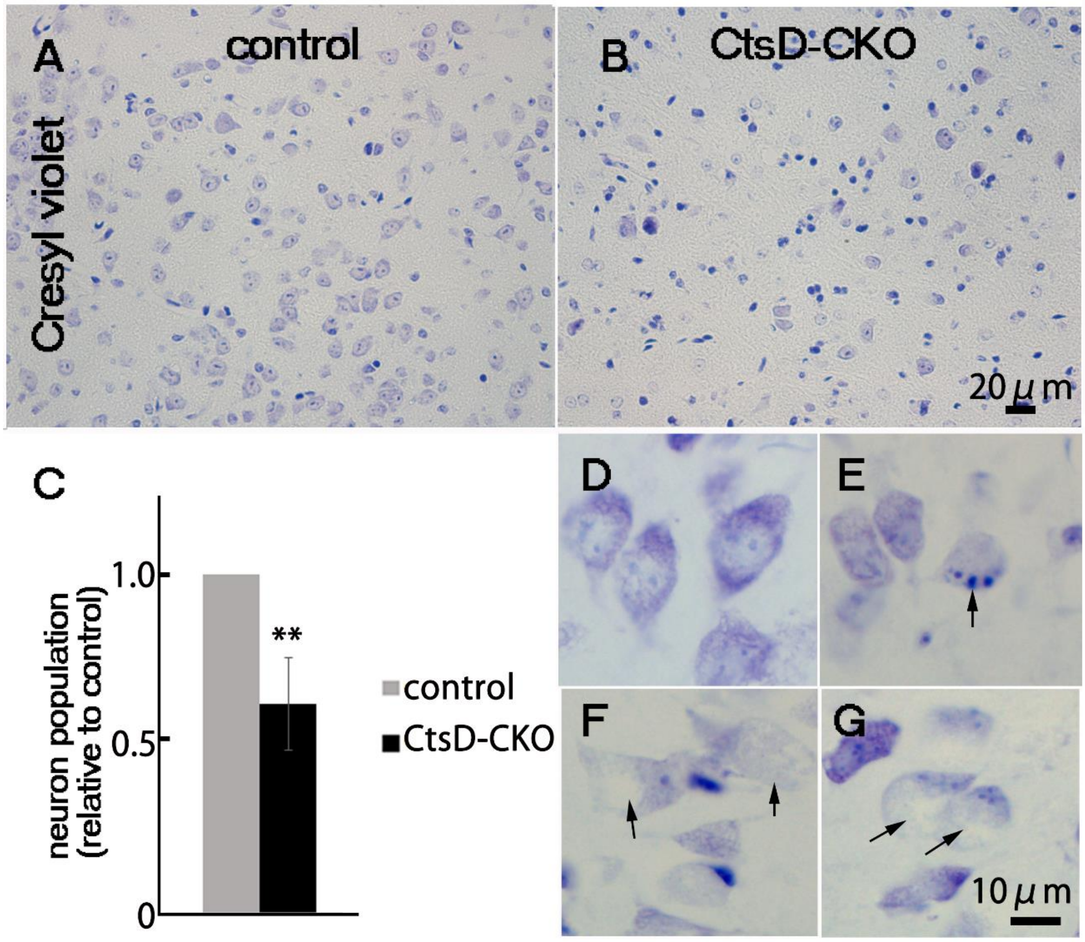

Supplementary Fig. 6. Cresyl violet staining in thalamus of CtsD-CKO and control mice. Representative images of thalamus of CtsD-CKO mice (B) and littermate control (A) at p32 were shown. Scale bar: 20 µm. In the thalamus of CtsD-CKO mice, the number of neurons was less than that of control littermates (C) (**$p<0.03$, from p19 to p32 mice, n=6). Neuronal cell possessing clear Nissl's body in control mouse (D), but in CtsD-CKO mouse there were abnormal neuronal cells with possessing deeply staining granules (E, arrow), ballooning cytoplasm (F, arrows), and ovoid shape cell soma with laterally displaced nucleus (G, arrows). Scale bar: 10 µm.

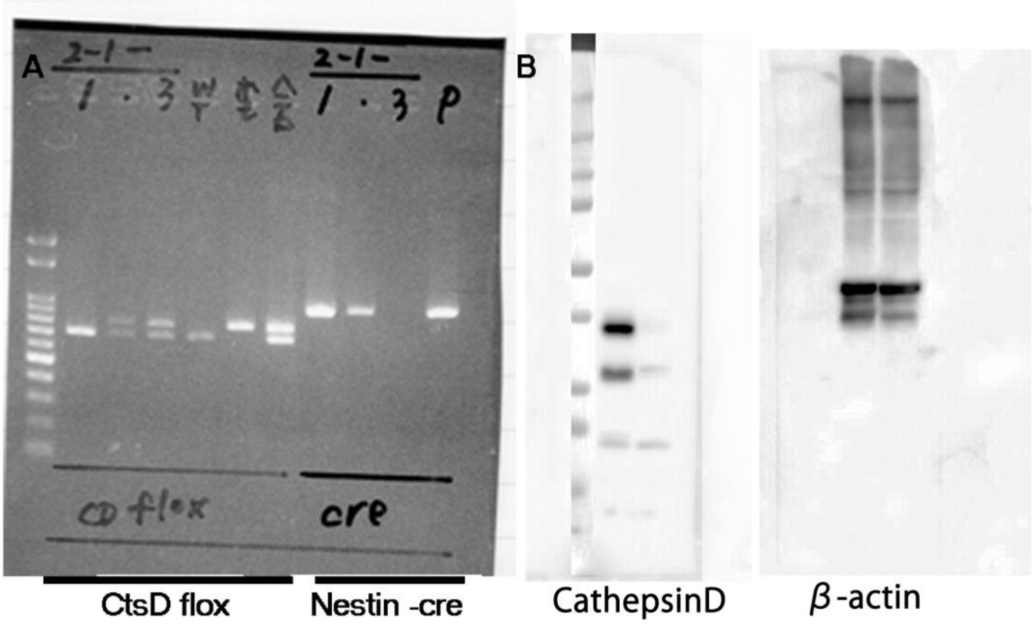

Supplementary Fig.7. Uncropped PCR and western blot images.

Uncropped images of the PCR data displayed in Figure 1A (A) and the western blots displayed in Figure 1B (B, * cathepsin D, ** procathepsinD) were shown. The same membrane was re-blotted with anti β-actin antibody. The dotted boxes indicated the areas shown in Figure 1A and 1B.
